# Misdiagnosed centipede and scorpion poisoning characterized by delayed hypersensitivity reaction: A case report

**DOI:** 10.1097/MD.0000000000032288

**Published:** 2022-12-23

**Authors:** Yaxin Li, Qiaoling Jin, Zhong Li, Menglin Chen, Linshen Xie

**Affiliations:** a West China School of Public Health and West China Fourth Hospital, Sichuan University, Chengdu, China; b West China-PUMC C.C. Chen Institute of Health, Sichuan University, Chengdu, China.

**Keywords:** allergic reaction, centipede, scorpion

## Abstract

Traditional Chinese medicine is widely used in China and Asian countries. According to the traditional Chinese medicine theory, centipedes and scorpions have the functions of relaxing spasm, eliminating masses, relieving pain, and dredging meridians and collaterals. Improper medication can lead to serious adverse reactions.

**Patient concerns:**

One 38-years-old female presented to our hospital because of cough and fever for more than 10 days. Ineffective anti-infection treatment, delayed skin rashes and supplementary medical history guided us to take centipede and scorpion poisoning into consideration.

**Diagnoses:**

Delayed hypersensitivity caused by centipedes and scorpions.

**Interventions:**

Anti-allergic therapy with glucocorticoid (methylprednisolone 40 mg/day) and *H*1 receptor antagonists (loratadine 10 mg/day).

**Outcomes:**

During the 1 year follow-up revealed, no fever, rash and any discomfort occurred.

**Lessons:**

This case suggests that because oral Chinese medicine poisoning is rare, detailed collection of medical history is particularly important for poisoning diagnosis.

## 1. Introduction

Traditional Chinese medicine (TCM) has a history of thousands of years in China and Asian countries. Centipede, scorpion, earthworm, cicada, snake and other animals are widely used because of their clinical efficacy and specific advantages. However, its adverse effects have been reported by more and more literatures, mainly focusing on liver, kidney, heart, nerve, carcinogenesis and gastrointestinal toxicity. It is well known centipede bite and scorpion sting can lead to neurotoxicity and hemolytic toxicity.^[1,2]^ In China, dead centipedes and scorpions are processed and taken orally as Chinese medicine. Oral poisoning of centipedes and scorpions is rarely reported and the risk is not recognized worldwide. Because people regard Chinese medicine as diet therapy or non-drug health care intervention, many patients do not take the initiative to mention the history of taking Chinese medicine when visiting a doctor, and doctors pay more attention to chemical drugs while ignoring the inquiry of Chinese medicine when investigating medical history. There are few reports of delayed hypersensitivity in oral poisoning of Chinese medicine. We report a case of centipede and scorpion poisoning characterized by delayed hypersensitivity.

## 2. Case report

The patient was a 38-years-old female with cough and fever for more than 10 days. She used to be in good health. Her past medical history includes breast hyperplasia surgery and cesarean section. She denied a history of allergies, infectious diseases, trauma, and exposure to industrial dust, radioactive substances and poisons. Systematic examination of all major organs and systems is not special. Physical examination at admission: body temperature 39.6℃, pulse 98/minutes, breathing 22/minutes, blood pressure 110/82 mm Hg, conscious, no yellowing of skin and sclera, no swelling of lymph nodes, no rash, normal jugular vein, low breathing sounds of both lungs without dry and wet rales, and no abnormality in other cardiopulmonary examinations. Moderate edema in lower limbs, with normal muscle strength and muscle tension, and negative pathological reflex signs.

**Figure 1.**
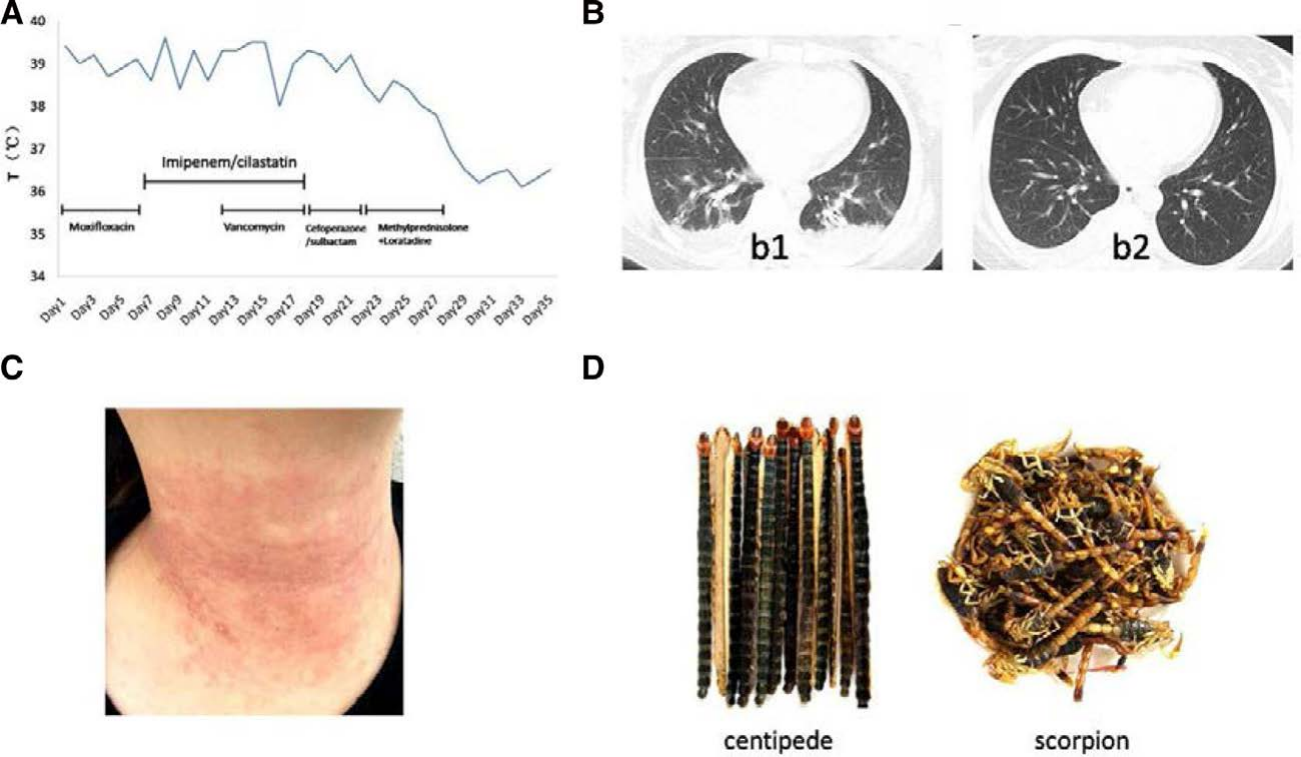
(A) The body temperature trend and the usage of various antibiotics. (B) Thoracic and abdominal CT: Multiple inflammatory exudations of bilateral lower lungs without any abdominal abnormalities on the first hospitalized day (b1).Obvious absorption of bilateral lower lung infection foci on the 14^th^ hospitalized day (b2). (C) Red rashes on this patient’s skin. (D) The centipedes and scorpions took by this patient. CT = computer tomography.

After being treated in the local hospital for 3 days, the patient was transferred to our hospital due to persistent fever. While searching for the cause of fever (lab results are shown in Table 1), she received anti-infection treatment (temperature and antibiotics are shown in Fig. 1A). Various antibiotics were used continuously, and antipyretics are used temporarily when the body temperature exceeding 39 ℃. On the 14th day of hospitalization, cough and dyspnea were obviously reduced, and computer tomography (CT) scan showed that the chest image of the patient improved (Fig. 1B). However, the fever lasted for more than 3 weeks, and the cause of fever was still unknown. On the 18th day of hospitalization, the patient’s limbs, shoulders, chest, back and neck developed several scattered red rashes without obvious itching. On the 21st day of hospitalization, the rash gradually became dense with itching, higher than the skin, and subsided by pressing (Fig. 1C). We organized relevant experts to consult and discuss. Radiologists and infectious disease experts believed that pulmonary infection has been controlled and inflammatory indicators tend to improve (White blood cell, erythrocyte sedimentation rate and Procalcitonin all drop to normal range after 7 days of treatment) by using a variety of powerful antibiotics. Therefore, the diagnosis of lung infection was difficult to fully explain the cause of fever. After consultation with experts in rheumatology, immunology and hematology, it was considered that the etiology of fever caused by tumor, immune and hematological diseases should be excluded according to the existing negative evidence. After poisoning experts carefully inquired about the medical history again, the patient recalled that she had taken centipede and scorpion orally (Fig. 1D) to treat lumbar disc herniation with TCM theory. She ground about 20 intact centipedes and 50 intact scorpions into powder and took 3 to 5 grams a day. She has difficulty in opening mouth, and felt numbness and swelling of extremities on the second day after taking centipedes and scorpions. However, these symptoms disappeared spontaneously on the fourth day so that the patient neglected these symptoms. After consulting poisoning experts, they considered that fever was a typical sign of delayed hypersensitivity caused by centipedes and scorpions. Antibiotics were canceled, and the patient was given methylprednisolone (40 mg × 3 days, then reduced to 20 mg × 3 days) combined with loratadine 10 mg daily for 6 days. On the third day, the patient’s body temperature quickly dropped to normal level (< 37 ℃). After 7 days, the rash disappeared completely. She was completely cured and discharged on the 35th day of hospitalization. Follow-up for 1 month, 3 months, 6 months and 1 year after discharge showed no fever, rash and any discomfort occurred.

**Table 1:** The key lab findings on admission.

Etiologic screening for fever		Results
Infectious Diseases	Regular blood test	Hemoglobin 86g/L; WBC 17.69 × 10^9^/L, Neutrophils 94.1%; ALB 26.3g/L; ESR 85.0mm/h; PCT 0.19ng/ml; Important markers of liver function, renal function, myocardial enzymes and coagulation function such as AST, ALT, Glucose, Urea, Creatinine, CK, PT, APTT were in normal range.
	Bacteria	Negative blood culture and sputum culture
	Fungus	Negative (1, 3)-beta-D glucan (G) test and galactomannan (GM) test in blood sample and alveolar lavage fluid
	Virus	Only the rhinovirus test was positive, the other test for EB-DNA, CMV-DNA, TORCH-IgM, EB virus antibody, influenza A H1N1, adenovirus, Boca virus, parainfluenza virus, hemipneumonia virus, influenza B virus, coronavirus, respiratory syncytial virus, hepatitis B, hepatitis C, HIV were negative.
	Tuberculosis	No acid-fast bacillus was found in sputum examination; T-SPOT and TB-IGRA test were negative, and TB-DNA in bronchoscopic alveolar lavage fluid was negative.
	Other pathogenic organisms	Negative antibodies against mycoplasma pneumoniae, chlamydia, treponema pallidum.
Malignant tumor		Tumor markers including CEA, CA125, CA199, NSE, AFP were in normal range; No malignant cells were found by thin liquid-based cytology in bronchoscopy brush.
Hematological system diseases		Immunofixation electrophoresis, serum protein electrophoresis, bone marrow aspiration smear and biopsy slice showed no significant abnormalities.
Rheumatism and immunology diseases		ANA, ENA antibody, immunoglobulin concentration (IgG, IgE, IgA, IgM, IgD), complements (C3, C4), circulating immune complex, T cell subsets (CD3, CD4, CD8), ANCA, IL-6 were in normal range.

AFP = alpha fetal protein, ALB = Albumin, ALT = alaninetransaminase, ANA = anti-nuclear antibody, ANCA = anti neutrophil cytoplasmic antibodies, APTT = activated partial thromboplastin time, AST = aspartate aminotransferase, CA125 = carbohydrate antigen 125, CA199 = carbohydrate antigen 199, CD3 = cluster of differentiation 3, CD4 = cluster of differentiation 4, CD8 = cluster of differentiation 8, CEA = carcinoembryonic antigen, CK = creatine kinase, CMV = cytomegalovirus, ENA = extractable nuclear antigen, ESR = erythrocyte sedimentation rate, HIV = human immunodeficiency virus, IgA = immunoglobulin A, IgD = Immunoglobulin D, IgE = immunoglobulin E, IgG = immunoglobulin G, IgM = immunoglobulin M, IL-6 = interleukin- 6, NSE = neuron-specific enolase, PCT = procalcitonin, PT = prothrombin time, TB = tuberculosis, TB-IGRA = tuberculosis -interferon gamma release assay, TORCH = toxoplasmagondi + rubella virus+ cytomegalo virus+ herpes virus, WBC = white blood cell.

## 3. Discussion

In the theory of TCM, centipede and scorpion have the functions of relieving spasm, reducing swelling, relieving pain and dredging meridians. Centipedes and scorpions have been used in China and some Asian countries to treat many diseases, such as stroke hemiplegia, epilepsy, stroke, whooping cough, tetanus, burns, tuberculosis and musculocutaneous diseases. Both of them can be used alone or in combination with compound prescription. In this case, centipedes and scorpions are used for oral administration.

It is well known thatcentipedes and scorpions are poisonous Chinese medicines. Centipede mainly contains 2 toxic components similar to bee venom, namely histamine-like substances and hemolytic protein, which can cause allergy, neurotoxicity, hepatotoxicity, cardiotoxicity and nephrotoxicity.^[3]^ Scorpion venom contains many small peptides and toxic proteins, and its main toxic component is neurotoxin, which has cholinergic and adrenergic effects. In addition, scorpion venom has hemolytic toxin, hemorrhagic toxin and thrombin.^[4]^ There are many reported cases of direct centipede bites and scorpion stings, and the toxic reactions and symptoms of poisoning are widely described, including local pain, swelling, congestion, paresthesia and systemic syndrome.^[4–7]^ In China, taking centipede and scorpion under the guidance of doctors has few adverse reactions caused by oral administration. Oral administration of centipedes and scorpions has been reported to cause progressive masseter myotonic spasm, neurotoxicity and anaphylactic shock,^[8,9]^ but no rash has been described. In this case, the patient decided to take centipedes and scorpions without medical guidance. We think that there are 2 reasons to explain the fever and rash in this patient: Generally, patient take the liquids after centipedes and scorpions been decocted in hot water in order to retain medicinal ingredients and destroy toxic ingredients. However, centipedes and scorpions powders were orally taken in this case, as a result, the toxicity of non-decocted centipedes and scorpions powder increased remarkably. According to the standard process of TCM treatment, centipede and scorpion can be ground into pills for oral administration, but the dosage is very low. The dose taken by this patient has exceeded the safe dose by more than 10 times.

In this case, neurotoxicity such as difficulty in opening mouth and numbness of limbs appeared on the second day but disappeared rapidly. At admission, the clinical manifestations were limited to pulmonary infection, mainly cough, sputum, dyspnea and CT findings. Because the neurotoxic symptoms were not serious and last for a short time, the patient did not provide the history of taking centipedes and scorpions, so it is difficult for doctors to consider poisoning. In the process of clinical diagnosis and treatment, bronchoscopy, bone marrow puncture and other examinations were performed according to the standard diagnostic procedure of uncertain fever. We tried to explain the patient’s fever with pulmonary infection, because the patient had typical symptoms, laboratory examination results (increased white blood cell count, Procalcitonin concentration, erythrocyte sedimentation rate level) and CT manifestations. Therefore, potent antibiotics include moxifloxacin, imipenem/cilastatin, vancomycin, cefoperazone/sulbactamwere chosen. However, the fever lasted for more than 20 days despite clinical symptoms and lab tests, CT manifestations showed the improvement of pulmonary infection. We did not know the history of taking centipedes and scorpions until her skin rashes were taken into serious consideration by specialists from department of poisoning medicine. The patient was finally diagnosed as delayed hypersensitivity secondary to centipedes and scorpions poisoning. According to the correct diagnosis, anti-allergic drugs such as methylprednisolone and loratadine showed good and rapid curative effect on fever and rash. Some studies showed that the incidence of rash in allergic reactions of Chinese medicine mostly occurs at the early stage,^[10]^ but the special feature of this case appeared at the later stage of the centipedes and scorpions poisoning.

In summary, this case suggests that patients should take Chinese medicine correctly to avoid poisoning. At the same time, clinicians should always keep in mind the importance of carefully inquiring about medical history (especially medication history) and pay attention to the toxic and side effects of Chinese medicine.

## Author contributions

**Conseptualization:** Yaxin Li.

**Supervision:** Linshen Xie.

**Writing – original draft:** Yaxin Li, Qiaoling Jin.

**Writing – review & editing:** Zhong Li, Menglin Chen, Linshen Xie.
